# Outcome of Renal Tissue Biopsy in Children and Adolescents Presenting with Features of Nephropathy at a Tertiary Hospital in Nigeria

**DOI:** 10.4314/ejhs.v35i2.4

**Published:** 2025-03

**Authors:** Olanrewaju Timothy Adedoyin, Olayinka Mikhail Buhari, Olayinka Rasheed Ibrahim, Olanrewaju Olubukola Oyedepo, Olusola Abidemi M Adesiyun, Abdurrazzaq Alege, Harrifatta Difirwiti

**Affiliations:** 1 Department of Pediatrics, University of Ilorin Teaching Hospital, Ilorin, Nigeria; 2 Department of Pathology, University of Ilorin Teaching Hospital, Ilorin, Nigeria; 3 Department of Pediatrics, Division of Clinical Medicine, University of Global Health Equity, Kigali, Rwanda; 4 Department of Anaesthesia, University of Ilorin Teaching Hospital, Ilorin, Nigeria; 5 Department of Radiology, University of Ilorin Teaching Hospital, Ilorin, Nigeria; 6 Department of Pediatrics, Federal Teaching Hospital, Katsina, Nigeria

**Keywords:** Biopsy, Oedema, Child, Nigeria

## Abstract

**Background:**

Oedematous renal lesions are significant chronic kidney diseases in childhood, with causes that may vary, especially in light of emerging illnesses like coronavirus, environmental pollution, and climate change. This study aimed to determine the histopathologic characteristics of oedematous renal lesions in children seen at a tertiary health facility in north-central Nigeria between January 2010 and December 2023.

**Methods:**

This was a prospective study conducted on all children aged 2-18 years who presented with features of oedematous renal lesions between January 2010 and December 2023 at a health facility in north-central Nigeria. All eligible patients underwent renal biopsy.

**Results:**

A total of 66 children with oedematous renal lesions were biopsied, comprising 35 males and 31 females, with a male-to-female ratio of 1.2:1. The age range of the subjects was 2-18 years, with a mean ± standard deviation (SD) of 7.8 ± 3.8 years. Of the 66 patients who consented to the biopsy, the histological findings were as follows: Minimal Change Nephropathy (MCNS)(n= 35, 53.0%), membranoproliferative glomerulonephritis (MPGN) (n=5, 7.6%), post-infectious glomerulonephritis (PSAGN) (n=3, 4.5%), and focal segmental glomerulosclerosis (FSGS)(n= 2, 3.0%).

**Conclusion:**

This study shows that the predominant histopathologic characteristic of childhood oedematous renal lesions was MCNS in the cohort of children studied.

## Introduction

Kidney disorders characterized by varying degrees of oedema are common among renal pathologies and contribute significantly to chronic kidney diseases (CKD) observed in children, particularly in tropical regions (1-2). Some oedematous renal lesions require renal biopsy for a definitive diagnosis. In cases of nephritic syndrome, renal biopsy is indicated if the clinical course is prolonged, particularly if there is normalisation of C3 and the presence of hypertension. Renal biopsy is an invasive procedure used to obtain renal tissue under ultrasound guidance for histopathological diagnosis. This study aimed to determine the outcomes of attempts to precisely diagnose renal lesions in children and adolescents who presented with varying features of nephropathy through renal tissue biopsy.

## Methods

This longitudinal study was conducted from January 1, 2010, to December 31, 2023, involving renal biopsies in children presenting with clinical features of nephropathies (nephrotic and nephritic syndromes) at the University of Ilorin Teaching Hospital, Ilorin, Nigeria. The University of Ilorin is a tertiary health facility in north-central Nigeria, with a dedicated pediatric nephrology unit responsible for managing children and adolescents with kidney-related disorders.

The study included children who presented with oedema and at least one of the following features: serum creatinine ≥106 µmol/l, haematuria, history of sore throat or skin rash, malar rash, hypertension, massive proteinuria (urinalysis of 3+ and above, protein/creatinine ratio > 2.0), serum cholesterol > 5.1 mmol/L, and serum albumin < 25 g/dl. Eligible patients who provided consent and assent after a detailed explanation of the procedure were assessed for suitability to undergo renal biopsy. Coagulation profiles were also performed, excluding patients with abnormal coagulation (platelet count < 90,000/uL, prothrombin time [PT] > 15 sec, partial thromboplastin time [PTT] > 45 sec, bleeding time > 5 min, and clotting time > 10 min) or a solitary kidney.

Eligible children underwent ultrasound-guided kidney biopsy at the lower pole of the kidney using a ‘Tru-cut’ disposable needle (Travenol) or 16-18G spring-loaded semiautomatic biopsy needles. Two kidney biopsy cores were taken from each patient for light microscopy. All patients tolerated the procedure well with no major complications. The kidney tissues were immediately examined under a microscope for the presence of glomeruli, then fixed in formalin, embedded in paraffin wax, and sectioned to 4 µm thickness. The sections were stained with haematoxylin and eosin (H&E), Periodic Acid Schiff (PAS), and Jone's methenamine silver. Histopathological characteristics were analyzed and reported by an experienced renal histopathologist and their team using light microscopy.

Descriptive statistical analysis was performed on the data. Age was summarized as mean ± standard deviation and further categorized into subgroups: under five, five to ten, and over ten years. The sex, histological characteristics, and outcomes were summarized using frequencies and percentages.

## Results

**Figure 1:**
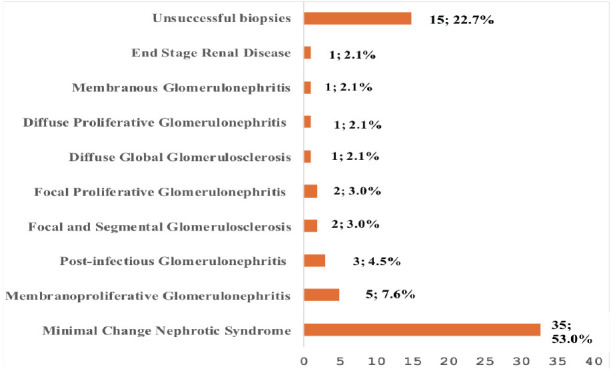
Histological characteristic of renal biopsy among the study children

A total of 66 children with features of nephrotic syndrome were biopsied [Table 1], comprising 35 males (53.0%) and 31 females (47.0%), with a male-to-female ratio of 1.2:1. The mean (SD) age of the cohort was 7.8 (3.8) years, with the majority (54.6%) aged between 5 and 10 years (36 children). The histological findings included Minimal Change Nephropathy (MCN) in 35 (53.0%) children, followed by membranoproliferative glomerulonephritis (MPGN) in 5 (7.6%), post-infectious glomerulonephritis (PIGN) in 3 (4.5%), and focal segmental glomerulosclerosis (FSGS) in 2 (3.0%) [Figure 1]. Fifteen biopsies (22.7%) were unsuccessful. Additionally, one child progressed to end-stage kidney disease (ESKD) during followup, with a case fatality rate of 1.5%.

**Table 1:** Characteristics of Childhood Oedematous Renal Lesions in the Study

Variable	n=66	Frequency	Percent
Age [Years]	Range	2-18	
	Mean (SD)	7.8 (3.8)	
	Less than five	14	21.2
	Five to ten	36	54.6
	Greater than ten	16	24.2
Sex	Male	35	53.0
	Female	31	47.0
Seasonal Pattern	Dry season (November-March)	22	33.3
	Wet Season (April-October)	44	66.7
Outcome	Alive	65	98.5
	Dead	1	1.5

## Discussion

Kidney disorders presenting with varying degrees of oedema are a leading cause of kidney morbidity and mortality in the tropics (1-2). Some patients do not require renal biopsy, but in cases of nephrotic-nephritic syndrome, where nephrotic range proteinuria is accompanied by features suggestive of nephritic syndrome (e.g., haematuria and hypertension), a renal biopsy may be necessary. In such cases, biopsy is performed due to the severity of clinical presentations. Adedoyin et al. (3) found that histopathological findings in such cases often indicate post-infectious glomerulonephritis. The most common finding in children with nephropathies in this study was Minimal Change Nephropathy (MCN), followed by membranoproliferative glomerulonephritis and post-infectious glomerulonephritis. MCN remains the most prevalent histopathological variant, as supported by other studies (4). Post-infectious glomerulonephritis commonly follows streptococcal infections, and renal biopsy is typically not indicated unless the course is prolonged or atypical, warranting further histopathological evaluation (5).

End-stage kidney disease (ESKD), observed in one case (2.1%) in this study, is not a typical indication for renal biopsy, as fibrosis in renal tissue contributes little to management. The biopsy was performed in this case because of steroid-resistant nephrotic syndrome and deteriorating renal function in the presence of ESKD.

Acute glomerulonephritis is a classic form of nephrotic oedema (4). Overexpansion of the vascular compartment contributes to the hemodynamic features of nephritic syndrome (increased effective arterial blood volume, plasma volume, blood pressure, and cardiac output). Hypervolemia and oedema result from primary salt retention and glomerular filtration rate (GFR) reduction due to glomerulopathy, which leads to urinary protein loss, increased tubular albumin reabsorption, and enhanced salt and water retention.

In conclusion, this study shows that Minimal Change Nephropathy (MCN) is the predominant histopathologic characteristic of oedematous childhood renal lesions. However, this conclusion is provisional, as histopathologic evaluation was based solely on light microscopy. A more definitive diagnosis could have been achieved with additional evaluations, including electron microscopy (EM) and immunofluorescence (IF), which were not available at our center.
